# The effect of two types of minimal acupuncture on stooling, sleeping and feeding in infants with colic: secondary analysis of a multicentre RCT in Sweden (ACU-COL)

**DOI:** 10.1177/0964528420920308

**Published:** 2020-05-06

**Authors:** Kajsa Landgren, Inger Hallström, Iren Tiberg

**Affiliations:** Department of Health Sciences, Faculty of Medicine, 5193 Lund University, Lund, Sweden

**Keywords:** acupuncture, feeding, infantile colic, randomized controlled trial, sleep, stooling

## Abstract

**Background:**

Evidence for the effect of minimal acupuncture in infants with colic is limited.

**Aim:**

To compare the effect of standardized minimal acupuncture, individualized acupuncture (where traditional acupuncture points were chosen according to the infant’s symptoms) and no acupuncture on objective measures of stooling, feeding and sleeping in infants with colic (based on diaries) and perceived changes in these parameters (based on parental questionnaires).

**Methods:**

This was a secondary analysis of a multicentre randomized controlled three-armed trial conducted in four counties in Sweden between January 2013 and May 2015 (ACU-COL). The effect on crying has already been published and showed a decrease in crying time for the acupuncture groups. Infants, 2–8 weeks old, who cried and fussed for more than 3 h/day for more than 3 days/week, and thereby fulfilled the criteria for infantile colic, received four extra visits to their ordinary child health centre. The infants (n = 147) were randomly allocated via a computer-generated list to standardized minimal acupuncture at LI4 for 5 s (group A, n = 48), semi-standardized individual acupuncture with a maximum of five insertions for up to 30 s (group B, n = 49), or no acupuncture (group C, n = 48). The parents and the ordinary staff were blinded. Data were collected using: (1) diaries at baseline, during the two intervention weeks and 1-week follow-up; and (2) questionnaires with quantitative and qualitative components used at the second and fourth visits and during a follow-up telephone call. Outcomes were the changes in frequency of stooling and in hours of sleep per day.

**Results:**

There were no differences between groups for stooling, feeding, or sleeping at any time point according to data from the diaries. At the follow-up phone call, more parents in groups A and B (compared to group C) perceived that feeding and sleep had changed and that the symptoms of colic had improved.

## Introduction

Acupuncture with strong stimulation affects gastrointestinal motility^1,2^ and promotes sleep^3^ in adults. In this article, the effect of two types of minimal acupuncture on stooling, sleeping and feeding patterns in infants with colic is explored.

Frequency of stooling, feeding, sleeping and crying in healthy infants has considerable variation between countries. Stooling is reported to occur 2.2–6 times/day when infants are 1 month old, decreasing to 1–2.2 times/day when they are 2 months old.^4^ Infants are fed a mean of eight times/day. They sleep about 13 h/day when they are 2–12 weeks.^5^ About 10% of newborns fuss and cry for more than 3 h/day on more than 3 days/week and are thereby defined as having infantile colic. Excessive crying has been linked to feeding problems and disturbed sleep.^6^

Acupuncture works through the autonomic system, relieving pain including visceral pain. Gastrointestinal motility is regulated by electroacupuncture stimulation of the abdomen, legs and arms, at least when combined with electrical stimulation in rats and mice with intact parasympathetic pathways.^1^ Few studies of acupuncture have been performed in infants.^7^ An earlier randomized controlled trial (RCT) including 90 infants with colic showed no major effect of minimal acupuncture on feeding, stooling and sleeping, although a minor effect on stooling and sleeping could not be ruled out.^4^

Furthermore, a case series including 913 children with colic indicated that the infants’ gastrointestinal problems were reduced after minimal acupuncture at LI4.^8^

Parents are often worried about their babies’ crying, stooling, feeding and sleeping. To be able to give comforting confirmation of normality, information about normal patterns is a valuable tool for health professionals in everyday clinical practice. It is also of interest to gain more knowledge about the effect of minimal acupuncture in infants and consider the issue of point specificity; traditional acupuncturists claim that the selection of traditional acupuncture points according to the patient’s symptoms and signs is vital. In a thorough review of the literature, however, some studies showed no difference in effect between acupuncture at verum and sham acupuncture points.^9^ The difference in effect of standardized minimal acupuncture at one single point versus minimal acupuncture at points chosen by an experienced acupuncturist according to symptoms in infants with colic, versus no acupuncture, was evaluated in the ACU-COL RCT. The primary outcomes – the child’s crying, adverse events and parents’ blinding – have been published.^10^ Crying was reduced in both acupuncture groups compared to the control group. In the present article, results of the secondary outcomes from ACU-COL will be presented. Thus, the aim of the part of the ACU-COL study presented in this article was to compare the effects of standardized minimal acupuncture, individualized acupuncture (where points were chosen according to the infant’s symptoms) and no acupuncture on the secondary outcomes of stooling, feeding and sleeping in infants with colic. A further aim was to describe perceived changes in the infants’ colic, stooling, feeding and sleeping.

## Methods

In a prospective, three-armed, randomized, controlled multicentre trial, infants with colic were randomized into three groups, receiving either minimal acupuncture at LI4 (group A), minimal acupuncture with 1–5 needles for 30 s (group B), or no acupuncture (group C). Quantitative data were captured in detailed diaries and parents rated changes in infants’ symptoms in questionnaires where they could also comment on the infant’s symptoms. Parents and the nurse they met at the child health centre (CHC) were blinded to the infants’ group allocation. The procedures are described in detail in the study protocol.^11^

### Participants and intervention

**Figure 1.**
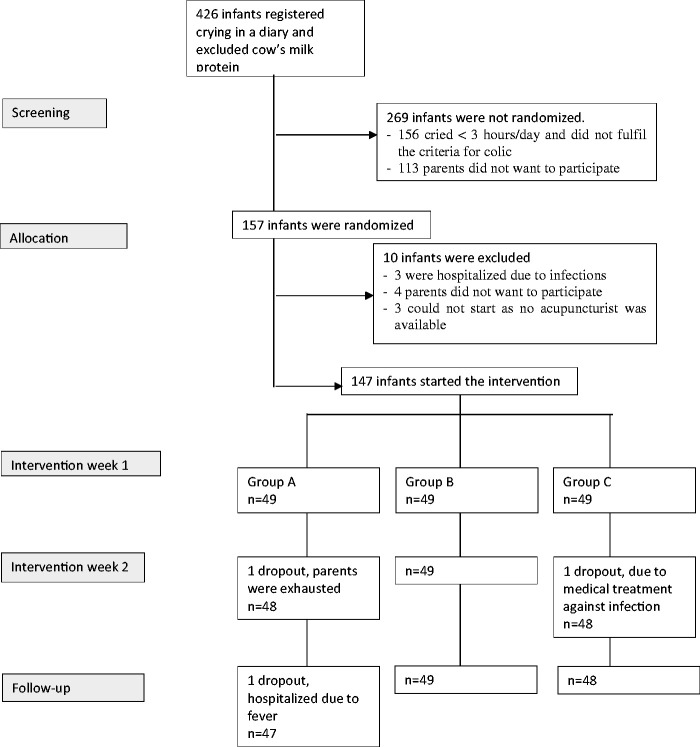
Flow chart of the study. Source: Adapted with permission from Landgren and Hallstrom.^10^

The study was conducted at four CHCs, of which two were situated close to the second and third biggest cities, and two in smaller towns, in Sweden. CHC offers a free-of-charge programme for all children. During the first 3 months, the programme includes seven visits where infants are weighed and measured and the parents receive routine childcare advice. All parents who could understand and read Swedish and were seeking help for excessively crying infants who were 2–8 weeks of age, and who had delivered at full term and were otherwise healthy, were informed about the trial. The 426 families who wanted to participate registered their infants’ crying, stooling, feeding and sleeping in a detailed diary for 1 week at baseline (BL). If they had not yet tried a diet excluding cow’s milk protein (in breast milk via the mother and/or formula) for at least 5 days, they did that during the BL week. Thus, infants who responded well to a cow’s milk–free diet were excluded. A total of 147 infants who, according to the diary, cried and/or fussed more than 3 h per day for more than 3 days at BL were included and randomized (Figure 1) via a computer-generated list by a project co-ordinator who informed the acupuncture nurse about group allocation. At BL, the infants had a mean age of 5 weeks and 58% were breastfed. For background data, see Table 1.

**Table 1.** Background data for participating infants.

	Group A^a^(n = 49)	Group B^b^(n = 49)	Group C^c^(n = 49)
Female, n (%)	26 (53)	20 (41)	21 (43)
Age at colic onset (weeks), median [IQR]	2.0 [1.0–2.0]	2.0 [1.0–3.0]	2.0 [1.0–3.0]
Age at inclusion (weeks), median [IQR]	6.0 [4.5–6]	6.0 [4.5–7]	6.0 [5.0–7.0]
Solely breastfed, n (%)	31 (63)	30 (61)	24 (49)
Maternal smoking, n (%)	0 (0)	2 (4)	1 (2)

Source: Adapted with permission from Landgren and Hallstrom.^10^

IQR: interquartile range.

^a^Group A received standardized minimal acupuncture at LI4 for 5 s.

^b^Group B received semi-standardized individual acupuncture with a maximum of five insertions for up to 30 s.

^c^Group C received no acupuncture.

Parallel to usual care at the CHC, infants in all groups came with a parent to four extra visits: twice a week for 2 weeks. Parents got evidence-based advice and support from the nurse, including encouragement for breastfeeding mothers to continue breastfeeding. The baby and the diary were then carried by the nurse to the acupuncturist, who was left alone with the baby and the diary for 5 min. The project leader had informed the acupuncturist about group allocation. Group A received the same type of acupuncture used in an earlier study:^12^ standardized acupuncture at LI4, unilaterally, on the right or left hand every second treatment. Needles were inserted to a depth of about 3 mm and retained for 2–5 s. In Group B, the acupuncturist was allowed to choose a total of 1–5 insertions at the traditional acupuncture points LI4, ST36 (unilaterally or bilaterally) or *Sifeng* (counted as four insertions) depending on the infant’s symptoms according to the diary. As the acupuncturists inserted the needles without or with only very light manipulation and did not seek *de qi* sensation, the stimulation was considered to be ‘minimal’ in both acupuncture groups. The needles used were 0.20 × 13 mm (Vinco, Helio, Jiangsu Province, China). Infants in Group C (control group) came four times to the CHC like the infants in Groups A and B, were treated in the same way and similarly spent 5 min with the acupuncturist but did not receive acupuncture.

As infants are involved in this research, careful ethical reflections were made.^11^ The Regional Ethical Review Board approved this study (ref. Dnr 2012/620). Both parents gave written consent before inclusion. The ACU-COL RCT was prospectively registered at ClinicalTrials.gov (ref. NCT01761331) on 4 January 2013.

### Data collection and analysis

Two instruments were used for data collection. The first, a detailed diary where the day and night were divided into 5-min intervals, was used by the parents at BL and during intervention week 1 (IW1), intervention week 2 (IW2) and the 3–6 follow-up days to register the duration of the infants’ crying, feeding and sleeping. Stooling was registered in the diary as either ‘large bowel movement’ or ‘small bowel movement’, defined as ‘a stain of faeces in the nappy’.

The second instrument was a structured questionnaire, managed by the ordinary CHC nurse, blinded to group allocation, at the second, third and fourth visits. Parents’ answers were either filled in by themselves or by the nurse. The same questions were asked by a project assistant, also blinded to group allocation, by telephone after the follow-up period. Questions were related to perceived changes in the infants’ symptoms, such as ‘Has the stooling of your infant changed, yes or no?’ and ‘Has your baby’s feeding habits changed, yes or no?’ Parents were also asked ‘Is your baby’s sleep much worse, worse, like before, better or much better?’ and ‘Has your baby’s colic changed? If so, it is much worse, worse, like before, better or much better?’ Parents were invited to comment on the infants’ symptoms using free text. These comments were recorded in the questionnaire by the parent or nurse at the visits to the CHC and by the project assistant at the follow-up call. Data were collected between January 2013 and May 2015.

Statistical analyses were conducted using the data collected in forms and records. Descriptive statistics are presented as n (%), mean ± standard deviation (SD), median [interquartile range (IQR)], or median (range). The data from the diaries were compared between the groups using analysis of covariance (ANCOVA) adjusted for BL values. Power was estimated based on the primary outcome of crying time, the results of which were reported in our previous article.^10^ Results are presented with 95% confidence intervals to show estimates and uncertainty. Differences between groups were assessed using Fisher’s exact test, Kruskal–Wallis test, or Mann–Whitney U test as appropriate. Pairwise comparisons between the groups were only performed if the overall ANCOVA test was significant. A two-sided p value <0.05 was considered statistically significant. All data analyses were performed using the Statistical Package for the Social Sciences (SPSS) version 24 for Windows (SPSS Inc., Chicago, IL, USA).

The comments written in free text in the questionnaire at visits 2, 3 and 4 and at the follow-up telephone call were summarized and analysed with manifest content analysis.^13^ The comments, often short, were coded according to content, and similar codes were grouped into categories. The number of times a code was included in a category was noted.

### Outcomes

Outcomes were change in frequency of stooling per day and change in hours of sleep per day at BL and during each of the two intervention weeks and the follow-up period, how parents rated changes in their infant’s colic, stooling, feeding and sleep in a questionnaire with quantitative and qualitative components.

## Results

According to parents’ reports in the diaries, infants in each group defecated a median 3.7 times/day (including small and large bowel movements) at BL. The frequency of bowel movement decreased to median of 1.8 times/day during follow-up. Infants in all groups were fed 8.7 times/day (mean), for a 166 min/day (mean; range 35–377) at BL. Mean feeding time decreased to 153 min/day (range 34–353) at follow-up (Table 2). Infants in each group slept for 710 min/day (mean) during baseline, increasing to 755 min/day (mean) during follow-up (Table 2). There were no differences either within groups or between groups for stooling, feeding, or sleep at any time point according to registration in the diaries.

**Table 2.** Frequency of stooling, feeding and duration of sleep.

	Group A^a^(n = 48)	Group B^b^(n = 49)	Group C^c^(n = 48)	p value, overall ANCOVA	Mean difference: A–C (95% CI)	Mean difference: B–C (95% CI)
Stooling, number of stools/day, median [IQR]						
Baseline	3.4 [1.3, 4.5]	4.0 [1.5, 5.8]	2.4 [1.0, 6.3]			
Intervention week 1	2.5 [1.2, 4.5]	2.9 [1.1, 5.6]	2.6 [0.8, 4.8]	0.888	−0.1 (−0.7, 0.4)	−0.1 (−0.6, 0.4)
Intervention week 2	2.4 [1.3, 4.2]	2.1 [1.0, 5.0]	2.4 [0.8, 4.0]	0.430	0.4 (−0.2, 1.0)	0.3 (−0.3, 0.9)
Follow-up	1.9 [0.8, 3.5]	2.0 [1.0, 5.0]	1.6 [1.0, 4.2]	0.580	0.1 (−0.5, 0.8)	0.3 (−0.3, 1.0)
Feeding, times/day, mean ± SD						
Baseline	8.5 [2.2]	9.0 [2.5]	8.5 [2.1]			
Intervention week 1	8.5 [1.7]	8.7 [2.1]	8.7 [2.2]	0.113	−0.3 (−0.7, 0.1)	−0.4 (−0.9, 0.0)
Intervention week 2	8.3 [2.0]	8.8 [2.4]	8.7 [2.3]	0.181	−0.5 (−1.0, 0.1)	−0.4 (−0.9, 0.2)
Follow-up	8.1 [1.9]	8.7 [2.4]	8.4 [2.1]	0.400	−0.4 (−0.9, 0.2)	−0.2 (−0.7, 0.4)
Sleeping, minutes/day, mean (SD)						
Baseline	723 [116]	705 [141]	700 [98]			
Intervention week 1	757 [110]	733 [148]	720 [91]	0.462	20 (−12, 51)	8 (−24, 39)
Intervention week 2	767 [139]	736 [150]	728 [96]	0.613	19 (−22, 61)	4 (−37, 45)
Follow-up	777 [121]	748 [144]	740 [92]	0.611	19 (−21, 58)	3 (−36, 42)

ANCOVA: analysis of covariance; CI: confidence interval; IQR: interquartile range; SD: standard deviation.

Data from the diaries were compared between the groups using ANCOVA adjusted for baseline values.

^a^Group A received standardized minimal acupuncture at LI4 for 5 s.

^b^Group B received semi-standardized individual acupuncture with a maximum of five insertions for up to 30 s.

^c^Group C received no acupuncture.

There were no differences between groups at any time point, whether or not parents perceived if stooling had changed according to the ‘yes’ or ‘no’ question. Likewise, there were no differences between groups in perceived changes in feeding at the first three time points. At the time of the follow-up phone call, significantly more parents in groups A and B rated that feeding had changed (Table 3). Pairwise comparisons between the groups showed that there was a larger proportion of changes in groups A and B than in group C (A vs C, p = 0.002; B vs C, p = 0.005; A vs B, p = 0.752). Likewise, significantly more parents in groups A and B perceived that sleep had changed (A vs C, p = 0.001; B vs C, p = 0.001; A vs B p = 0.92) and that the symptoms of colic had improved (A vs C, p < 0.001; B vs C, p < 0.001; A vs B, p = 0.157) at the time of the follow-up phone call (Table 3).

**Table 3.** Results of questionnaires.

		Group A^a^(n = 48)	Group B^b^(n = 49)	Group C^c^(n = 48)	Overall p value
Change in stooling, “yes” responses, n (%)^d^	Visit 2	23 (47%)	16 (33%)	20 (41%)	0.351
	Visit 3	26 (55%)	20 (41%)	22 (46%)	0.353
	Visit 4	30 (63%)	28 (57%)	26 (54%)	0.704
	Follow-up^e^	27 (56%)	33 (67%)	25 (52%)	0.287
Change in feeding, “yes” responses, n (%)^d^	Visit 2	12 (25%)	13 (27%)	19 (39%)	0.248
	Visit 3	22 (46%)	21 (43%)	19 (40%)	0.826
	Visit 4	24 (50%)	20 (41%)	19 (40%)	0.530
	Follow-up^e^	27 (56%)	26 (53%)	12 (25%)	0.003^f^
Sleeping, median [IQR]^g^1 = Much worse2 = Worse3 = Like before4 = Better5 = Much better	Visit 2	3 [3, 4]	3 [3, 4]	3 [3, 4]	0.521
	Visit 3	4 [3, 4]	4 [3, 4]	3 [3, 4]	0.026
	Visit 4	4 [3, 4]	3 [3, 4]	3 [3, 4]	0.093
	Follow-up^e^	4 [3, 4]	4 [3, 4]	3 [3, 4]	0.001^f^
Colic, median [IQR)^g^1 = Much worse2 = Worse3 = Like before4 = Better5 = Much better	Visit 2	4 [3, 4]	4 [3, 4]	4 [3, 4]	0.480
	Visit 3	4 [4, 4]	4 [3, 5]	4 [3, 4]	0.338
	Visit 4	4 [4, 5]	4 [4, 5]	4 [3, 4]	0.069
	Follow-up^e^	5 [4, 5]	5 [4, 5]	4 [3, 4]	<0.001^f^

IQR: interquartile range.

The following questions were posed: • ‘Has your baby’s stooling changed, yes or no?’• ‘Has your baby’s feeding habits changed, yes or no?’• ‘Is your baby’s sleep much worse, worse, like before, better or much better?’• ‘Have the symptoms of your baby’s colic changed? If so, are they much worse, worse, like before, better or much better?’

^a^Group A received standardized minimal acupuncture at LI4 for 5 s.

^b^Group B received semi-standardized individual acupuncture with a maximum five insertions for up to 30 s.

^c^Group C received no acupuncture.

^d^Chi-squared test.

^e^Follow-up via telephone.

^f^Pairwise comparisons between groups showed a larger proportion of changes in groups A and B than in group C.

^g^Kruskal–Wallis test.

In the open-ended questions, parents commented on infants’ stooling 316 times, on infants’ feeding 256 times and on infants’ sleep 204 times. Codes and categories from all time points are shown in Table 4, which also shows examples of comments in each category and how many times a code was presented in each group.

**Table 4.** Codes deriving from parents’ comments on infants’ stooling, feeding and sleep.

Categories	Group A^a^(n = 48)	Group B^b^(n = 49)	Group C^c^(n = 48)
**Comments on infants stooling**			
Changes in effort			
Stooling is easier/better/without fussing and crying	15	18	5
Easier to pass wind	7	15	2
Stooling is worse/more difficult		3	3
Changes in frequency			
Less often	30	38	36
More often	25	18	20
Irregular	1		4
More regular	1	1	2
Changes in size			
Bigger	37	27	25
Smaller	2	2	2
Changes in texture			
Firmer consistency	3	4	2
Looser consistency	6	5	7
Altered consistency, including more or less slimy, smoother and “normalized”	9	6	6
Changes in colour			
Stools are green	4	3	3
Change in colour from green	5	1	4
Changes in odour			
Less smelly		4	
More smelly			5
**Comments on infants’ feeding**			
*Positive comments*: Eats better, eats more, more calmly, more regularly. More concentrated when eating. Less panic, not so voracious. Less comfort feeding. Better appetite, good appetite, more breastfeeding now, solely breastfed now, less formula.	90	98	31
*Negative comments*: Less breastfeeding. Feeding with formula more often. Switching from one formula to another. More difficult to feed. Bad appetite. Comfort feeding, eats very frequent. Irregular feeding. Fussy when being fed.	21	13	57
**Comments on infants’ sleep**			
*Positive comments*: Sleeps longer periods. Deeper sleep. Falls asleep more easily. Can sleep in own bed. Sleeping without restlessness and fussing. Lies still when asleep. Has slept a whole night. Sleeps better during the daytime.	61	53	26
*Negative comments*: Difficult to calm down. Disturbed sleep. Sleeps only a few minutes. Splashes and whines while asleep. Wakes up often. Preferably sleeps in your arms.	16	12	22
*Neither positive nor negative*: Sometimes better sleep, sometimes worse.	3	7	10

CHC: child health centre.

Data derived from free text in the questionnaires from the second, third and fourth visits to the CHC and from the follow-up phone call, with examples of comments in each category. Numbers represent how many times this code was expressed by parents in each group.

^a^Group A received standardized minimal acupuncture at LI4 for 5 s.

^b^Group B received semi-standardized individual acupuncture with a maximum of five insertions for up to 30 s.

^c^Group C received no acupuncture.

## Discussion

As gastrointestinal symptoms in infants are the most common reason for parents to seek paediatric health care,^14^ knowing ‘normal’ patterns facilitates giving reliable advice. The frequency of stooling in the present study was the same as in a cohort of healthy breastfed infants in the Netherlands, a country with similar living conditions, in which the frequency decreased from 3.7 to 1.9 times per day between the first and the third months.^15^ In our study, both small and large bowel movements were registered, and only 58% were breastfed (a factor known to increase stooling frequency) compared to the 100% reported in the aforementioned Dutch study by den Hertog et al.^15^ This supports the findings of Tunc et al.^16^ who found less frequent stooling in infants with colic compared to infants without colic. The decrease over time was expected.^15^ Infants in the present study slept an hour less than healthy infants,^5^ who slept for 13.2 h/day. The infants were reportedly fed 8.5 times/day (median) following international recommendations, with a wide variation between 0.5 and 6.5 h/day. However, only 58% were breastfed, compared to the national level of 84%, indicating that colic may disturb breastfeeding.

The diaries showed no statistically significant differences between infants who received either of the two types of minimal acupuncture and infants who did not get acupuncture, indicating that acupuncture with light stimulation does not affect the frequency of infants’ stooling or sleep, in contrast to studies showing effects in adults with stronger stimulation.^1,3^ Furthermore, there were no differences between the two groups receiving minimal acupuncture, suggesting that neither the choice of traditional acupuncture points nor the number of insertions (one vs up to five insertions) or duration of needling (5 vs up to 30 s) made a significant difference in this context.

According to the diaries, minimal acupuncture did not influence the frequency of stooling, feeding and sleep. More parents answered ‘yes’ to the questions regarding whether these behaviours had changed but, from this dichotomous question, we do not know whether the change was perceived as positive or negative. However, parents’ subjective descriptions of their infants’ symptoms were more positive in the acupuncture groups than in the control group. Parents who perceived the stooling to increase or decrease were equally distributed between groups, which follows the recordings in the diaries and the fact that parents in all groups commented that the size of the stools increased, which is expected as infants grow older. However, apparently more parents in the acupuncture groups perceived a difference in the infants’ stooling, described as ‘stooling is easier’ and ‘easier to pass wind’, suggesting an effect that was not captured in measures of frequency. Divergent findings when results from quantitative and qualitative methods are integrated are common and interesting, showing that the methods capture different aspects of the topic, such that a more nuanced understanding may be achieved.^17^ In the present study, divergent findings were also found regarding feeding and sleep. Although there were no differences between groups in the frequency and duration of feeding, neither in this nor in an earlier study,^4^ parents of infants who received acupuncture in the present study evidently provided more positive comments on feeding, such as ‘eats better’, ‘eats more calmly’ or ‘eats more regularly’, compared to parents in group C, who more often provided negative comments like ‘more difficult to feed’. These subjective reports of perceived positive changes are in line with a retrospective study performed in a neonatal intensive care unit (NICU) that described 10 infants who received acupuncture for feeding problems and agitation,^18^ with a case report including 913 infants who had received minimal acupuncture for gastrointestinal symptoms,^8^ and with an earlier trial where infants with colic received minimal acupuncture.^4^ In the latter, parents of infants in the acupuncture group commented on altered and normalized stooling in their infants, described fewer problems with feeding and reported improvement of colic and sleep more frequently than the control group, in line with the present study.

Significantly more parents in the acupuncture groups reported that their infants’ symptoms of colic were improved. This is in line with our previously published results that showed in the same cohort of infants that crying was reduced more in the acupuncture groups (groups A and B) than in group C, which did not receive acupuncture.^10^ However, a later systematic review including an individual patient data-analysis of three similar studies found that minimal acupuncture reduced crying time by only 25 min, which was less than the 30 min stipulated to be a clinically relevant difference for this parameter.^19^

The predominant symptom of colic is crying, which affects both sleep and feeding in a negative way.^6^ It is difficult to feed a crying baby, and parents may try to reduce crying by comfort feeding. The subjective positive outcomes in our present and previous^10^ reports might reflect that even a smaller reduction in crying, and small changes in frequency of stooling, feeding and sleep, may have reduced the infants’ symptoms to a degree where parents experience a valuable improvement. Qualitative nuances like ‘normalized stooling’, ‘can sleep in own bed’ and ‘eats more calmly, has less panic, is not so voracious’ imply a possible positive impact of acupuncture on outcomes other than time reduction or frequency. A systematic review^19^ examining the blinding of the parents using a diary to assess infants’ symptoms in a prior study by Landgren et al.^12^ and those in the acupuncture groups of ACU-COL^10^ considered them to be unblinded, as more parents in the acupuncture groups guessed that their infant had received acupuncture. The potential risk of bias when outcome assessors are not blinded should be considered, as well as the potential for them to ignore or disregard a real effect due to perceived unblinding of the participants.

As reported side-effects were mild, few and transient,^10^ and as conventional best practice offers no effective and safe treatment,^6^ acupuncture might be used to reduce symptoms in infants with colic during a period that is stressful for the entire family.^20,21^

### Strengths and limitations

This report follows the Consolidating Standards of Reporting Trials (CONSORT) guideline. Careful registration and follow-up of infants’ symptoms in a diary that has been used in earlier trials^4,12,10^ gave detailed information, which is a strength. Several variables and time points gave rich data but multiple comparisons increase the risk of false-positive findings.

A weakness of the study is that the parents’ subjective assessments were registered using diaries, opening up the possibility that other factors may have influenced the parents’ experience of the situation. Another weakness is that we did not control whether the comments followed the diaries, or whether the frequency of stooling changed in the desired direction of effect, mirroring the results of a review that concluded that acupuncture has a dual regulatory effect, manifest by the promotion of gastric peristalsis when gastric motility is low and suppression of peristalsis when motility is overactive.^1^ Furthermore, we did not monitor breastfeeding mothers’ diet besides the inclusion criterion of having tried at least 5 days without cow’s milk protein. This is a limitation as maternal diet affects breastfed infants.^22^ Likewise, bottle-fed infants might have switched formula during the study period. Thus, we cannot draw any conclusions regarding the potentially confounding effect of diet.

The effect of acupuncture depends on ‘dosage’ including degree of stimulation,^23^ and it is controversial whether the effect of acupuncture depends on needling location.^1,9^ Few studies have compared the effect of standardized acupuncture at a single traditional acupuncture point with individualized acupuncture. In this trial, standardized acupuncture comprising a single-needle insertion for 5 s, individualized acupuncture with 1–5 needle insertions for up to 30 s, and no acupuncture were compared. Thus, both acupuncture groups received minimal acupuncture compared to most trials/experiments in adults or animals, and the results cannot be extrapolated to other acupuncture styles with stronger degrees of stimulation.

In conclusion, this study shows how stooling, feeding and sleep were reported in a cohort of 147 infants with colic and thereby adds to a better understanding of infants who cry excessively. This may help professionals give more reliable advice about ‘normal’ patterns of stooling, sleep and feeding. The study found no differences between the acupuncture groups and the control group in frequency of stooling, feeding and sleep and no difference between the two types of minimal acupuncture. We do not know whether acupuncture at other sites (e.g. different traditional acupuncture points), delivered with stronger stimulation and/or more frequently, would have an effect. However, the qualitative data showed that parents of infants who received minimal acupuncture more often subjectively reported that the colic had improved and described positive changes in symptoms other than frequency. When quantitative and qualitative methods are combined, different findings and experiences may be captured, which can help inform future research directions.
